# New Insights into the Host–Pathogen Interaction of *Mycoplasma gallisepticum* and Avian Metapneumovirus in Tracheal Organ Cultures of Chicken

**DOI:** 10.3390/microorganisms9112407

**Published:** 2021-11-22

**Authors:** Nancy Rüger, Hicham Sid, Jochen Meens, Michael P. Szostak, Wolfgang Baumgärtner, Frederik Bexter, Silke Rautenschlein

**Affiliations:** 1 Clinic for Poultry, University of Veterinary Medicine Hannover, 30559 Hannover, Germany; nancy.rueger@tiho-hannover.de (N.R.); frederik.bexter@tiho-hannover.de (F.B.); 2 Reproductive Biotechnology, TUM School of Life Sciences Weihenstephan, Technical University of Munich, 85354 Munich, Germany; hicham.sid@tum.de; 3 Institute for Microbiology, Centre for Infection Medicine, University of Veterinary Medicine Hannover, 30559 Hannover, Germany; Jochen.Meens@tiho-hannover.de; 4 Institute of Microbiology, Department of Pathobiology, University of Veterinary Medicine Vienna, 1210 Vienna, Austria; Michael.Szostak@vetmeduni.ac.at; 5 Department of Pathology, University of Veterinary Medicine Hannover, 30559 Hannover, Germany; Wolfgang.Baumgaertner@tiho-hannover.de

**Keywords:** avian metapneumovirus, *Mycoplasma gallisepticum*, TOC, co-infection, innate immunity, interferon

## Abstract

Respiratory pathogens are a health threat for poultry. Co-infections lead to the exacerbation of clinical symptoms and lesions. *Mycoplasma gallisepticum* (*M. gallispeticum*) and Avian Metapneumovirus (AMPV) are two avian respiratory pathogens that co-circulate worldwide. The knowledge about the host–pathogen interaction of *M. gallispeticum* and AMPV in the chicken respiratory tract is limited. We aimed to investigate how co-infections affect the pathogenesis of the respiratory disease and whether the order of invading pathogens leads to changes in host–pathogen interaction. We used chicken tracheal organ cultures (TOC) to investigate pathogen invasion and replication, lesion development, and selected innate immune responses, such as interferon (IFN) α, inducible nitric oxide synthase (iNOS) and IFNλ mRNA expression levels. We performed mono-inoculations (AMPV or *M. gallispeticum*) or dual-inoculations in two orders with a 24-h interval between the first and second pathogen. Dual-inoculations compared to mono-inoculations resulted in more severe host reactions. Pre-infection with AMPV followed by *M. gallispeticum* resulted in prolonged viral replication, more significant innate immune responses, and lesions (*p* < 0.05). AMPV as the secondary pathogen impaired the bacterial attachment process. Consequently, the *M. gallispeticum* replication was delayed, the innate immune response was less pronounced, and lesions appeared later. Our results suggest a competing process in co-infections and offer new insights in disease processes.

## 1. Introduction

Although preventive measures and treatment options are available, respiratory diseases in poultry still cause high economic losses worldwide [1,2]. Different factors play an important role in the outcome, such as the type of husbandry, the environmental conditions, the immunological state of the birds, and, most importantly, the infecting agents. Further, the presence of multiple pathogens might have an impact on the course and severity of the respiratory disease [3,4,5,6,7]. Field and experimental studies have already described the correlation between co-infections and effects on the immune system, as well as clinical signs and pathological lesion development [3,4,7,8,9,10,11,12]. Two of these respiratory pathogens are avian metapneumovirus (AMPV) and *Mycoplasma gallisepticum* (*M. gallispeticum*), which (co-) circulate in poultry flocks worldwide [2,3,5,13,14,15]. While AMPV leads to avian rhinotracheitis (ART) and can cause swollen head syndrome (SHS) in chickens or turkey rhinotracheitis (TRT) in turkeys, *M. gallispeticum* induces chronic respiratory disease (CRD) [16]. Although it has been demonstrated how viruses and bacteria interact with the host [3,4,17,18,19], the knowledge of the host–pathogen–pathogen interaction of AMPV and *M. gallispeticum* in the respiratory tract is very limited. So far, one dual-infection study in turkeys involving AMPV and *M. gallispeticum* demonstrated a higher incidence of gross lesions during co-infections [7].

During mono-infections, both AMPV and *M. gallispeticum* show a similar tropism for ciliated cells of the respiratory tract [20,21]. By attaching to the cilia and apical border of the epithelium, they might evade the mucociliary clearance as a first defense mechanism. A successful attachment may be later followed by cell invasion [22,23]. While AMPV enters the cells by means of envelope-fusion with the cell membrane [24], *M. gallispeticum* accomplishes the invasion via penetration [22,25,26]. Interestingly, *M. gallispeticum* can reside intracellularly, resulting in a chronic or latent course of the disease [22,27]. In contrast, AMPV is hardly detectable when clinical symptoms occur [28,29]. The invasion process then activates innate immune responses, such as toll-like-receptor (TRL) -mediated cytokine production [27,30,31,32,33,34]. In vivo studies revealed insights into the cytokine and chemokine activation upon mycoplasma infection, such as the upregulation of interferon (IFN) α and IFNγ expression, as well as the modification of different toll-like-receptor (TLR) downstream pathways [35,36,37]. An in vitro study conducted with *M. gallispeticum*-infected tracheal epithelial cells revealed contrary results, with no significant effects on the IFNα and IFNλ expression levels in the chicken cells [4]. Studies with respiratory viruses, such as influenza virus, human respiratory syncytial virus, and human metapneumovirus (HMPV), demonstrated upregulations of IFNα and IFNλ in vivo and in vitro [32,38]. The results of AMPV infection studies depended on whether they were conducted in vivo or in vitro [21,39]. They found a downregulation of IFNα in vivo and upregulations of total interferon titer as well as an upregulation of nitric oxide (NO) production in vitro. All these studies pointed out that both *M. gallispeticum* and AMPV might induce pro-inflammatory processes, leading to focal ciliary loss, a subsequent decrease in the mucociliary clearance [21,40], and a consequently higher risk of secondary infections. It remains unknown how *M. gallispeticum* and AMPV interact with each other and with the host.

In our study, we used tracheal organ cultures (TOC) as an in vitro infection model. This organ culture provides a complete network of differentiated cells, and thus, many aspects of the innate immune response can be examined. The advantages of in vitro models, such as the reproducibility, comparable target tissue size, and reduction of animal experiments due to efficient use of material, have been demonstrated in various studies [41,42]. Moreover, the survival time of TOCs can be up to three or four weeks [43].

Our objectives for this study were to compare the host–pathogen interactions of AMPV and *M. gallispeticum* during mono- and co-infections of chicken TOCs. We did this by focusing on the colonization and replication of the pathogens, the microscopic lesion development, and the cytokine mRNA expression of the host, specifically the potent antiviral factors IFNα [44] and IFNλ [32,45], as well as inducible nitric oxide synthase (iNOS), known to be induced by viral and bacterial infections [46,47]. Furthermore, we wanted to determine the impact of the order of invading pathogens on the host immune response and pathogen replication. The results of this study will provide a better understanding of the infection and interaction mechanisms during an AMPV and *M. gallispeticum* co-infection, and therefore, they may help to develop and improve prophylactic measures and treatment options.

## 2. Materials and Methods

### 2.1. Tracheal Organ Culture

The preparation of the tracheal organ cultures (TOC) has been described previously [43]. Briefly, tracheae were dissected from humanely sacrificed 20-day-old specific-pathogen-free (SPF) chicken embryos (Valo BioMedia GmbH, Osterholz-Scharmbeck, Lower Saxony, Germany). They were subsequently cut into equal rings of 0.8 mm and placed separately into 5mL-tubes (Sarstedt AG & Co KG, Nümbrecht, North Rhine-Westphalia, Germany) filled with 1 mL pre-warmed (~37 °C) 199 Hanks’ salts medium (Sigma-Aldrich, Taufkirchen, Bavaria, Germany), supplemented with 1% L-glutamine (200mM, Biochrome, Berlin, Germany) and 1% Penicillin G (10,000 U/mL, Biochrome, Berlin, Germany). The TOCs were placed in an overhead shaker and incubated at 37 °C for four days. After microscopical evaluation, tracheal rings with 100% ciliary activity were randomly assigned to different groups.

### 2.2. Virus and Bacterium

The avian metapneumovirus (AMPV), Subtype B, Italy strain RF 63, kindly provided by Dr. C. Jones (University of Liverpool, UK), was propagated and titrated in TOCs as described previously [48]. The titer was calculated using the Reed and Muench method [49]. For each inoculation, a virus concentration of 1 × 10^3^ of 50% ciliostatic dose (CD_50_)/mL was used. The *Mycoplasma gallisepticum* (*M. gallispeticum*) S6 laboratory strain (in-house strain collection, stored in Frey’s medium at −80 °C) was adapted to and passaged in SP4 medium three times. The bacterial concentration was determined via the colony forming unit (CFU) assay [50]. TOC inoculations were performed with a bacterial dose of 1 × 10^4^ CFU/mL.

### 2.3. Experimental Design

Seven experiments were conducted to quantify the viral and bacterial replication (Experiment 1a and 1b), to detect their colonization pattern at the epithelial surface (Experiment 1c), and to examine the development of cytopathological lesions after TOC inoculation (Experiment 2a and 2b). Furthermore, the involvement of selected innate immune response parameters was investigated (Experiment 3a and 3b) (Table 1).

Unless otherwise stated, the following inoculation scheme was used for each experiment. Negative control TOCs were treated only with pathogen-free growth medium (group: control), *M. gallispeticum*-inoculated (group: *M. gallispeticum*) and AMPV-inoculated TOCs (group: AMPV), TOCs inoculated with *M. gallispeticum* and subsequently 24 h later with AMPV (group: *M. gallispeticum*/AMPV), and in reverse order (group: AMPV/*M. gallispeticum*).

The pre-experiment examination of bacterial replication in organ culture medium without TOCs was performed via CFU-Assay. The trial consisted of two groups (10 replicates/group) of organ culture medium inoculated with *M. gallispeticum*. To one of the groups, we additionally added one TOC per replicate. At 1 h post inoculation (hpi) and 49 hpi, the supernatant of five samples per group per time point were collected. Serially diluted supernatants were plated on Frey-Agar (in triplicates) and incubated under humid and microaerophilic conditions for seven days. Colonies were counted microscopically, and the average of the colony numbers per dilution was used to calculate the bacterial concentration (CFU/mL).

Experiment 1a: As indicators for pathogen replication, the viral and bacterial genomes were quantified. Inoculated and negative control TOCs *(n* = 5/group/time point) were collected at 1, 25, 49, 73, and 97 hpi and were further processed for RNA/DNA isolations and subsequent reverse transcriptase quantitative polymerase chain reactions (RTqPCR) (AMPV) and qPCR (*M. gallispeticum*).

Experiment 1b: Five TOCs/group/time point were embedded in paraffin wax and immunohistochemically processed for the detection of pathogen antigens on the epithelial surface.

Experiment 1c: Electron microscopy was performed to highlight the ultrastructure of the epithelial surface during bacterial and viral colonization 1, 25, 49, and 73 hpi. Additionally, further mono-infection time points were analyzed: 3, 7, and 13 hpi and 1-, 15-, 30-, and 45-min post inoculation (mpi).

Experiment 2a: Ten TOCs each were randomly allocated to five different inoculation groups. The inoculum consisted of virus or bacterium, diluted in 100 µL 199 Hanks’ salts medium. After each inoculation, TOCs were incubated at 37 °C for 1 h. Afterwards, 900 µL of pathogen-free growth medium, supplemented with 1% L-glutamine (200mM, Biochrome, Berlin, Germany), 1% Penicillin G (10,000 U/mL, Biochrome), and 0.2% bovine serum albumin (Carl Roth^®^, Karlsruhe, Germany) were added. The percentage of ciliary activity was assessed daily via inverted microscope (Zeiss, Germany) for ten days post-inoculation (dpi) as described before [44].

Experiment 2b: To assess lesion development, TOCs were embedded in paraffin wax and processed further for histopathological examination (with *n* = 5 / group / time point).

Experiment 3a included the detection of interferon alpha (IFNα), inducible nitric oxide synthase (iNOS), and interferon lambda (IFNλ) mRNA expressions in mono- and dual-inoculated groups compared to the negative controls *(n* = 5/group/time point). Samples taken at 1, 25, 49, and 73 hpi were processed for RTqPCR.

Experiment 3b: Furthermore, mRNA expression of IFNα were investigated at 1, 3, 5, 13, and 25 h post AMPV or *M. gallispeticum* inoculation.

All the described experiments were conducted separately and repeated at least once. The results represent summarized data from all repeated experiments.

#### 2.3.1. RNA and DNA Isolation

RNA/DNA isolation for pathogen quantification by (RT)qPCR was performed following the manufacturer’s instruction guide (RNA/DNA purification kit, KYLT^®^, AniCon, Germany). Standard curves of the pathogen stocks and negative TOCs were generated to verify the quality of the isolation procedure. The RNeasy plus mini kit (Qiagen, Hilden, North Rhine-Westphalia, Germany) was used for RNA isolations to determine the mRNA cytokine expression according to the manufacturer’s instructions. Spectrometric measurement of RNA purity and concentration was performed via NanoDrop (Spectrophotometer ND-1000, peqlab, Biotechnologie GmbH, Erlangen, Bavaria, Germany).

#### 2.3.2. Quantification of Pathogens by (RT)qPCR

Primers and probes are listed in Table S1. (RT)qPCR was conducted using Quanta qScript™ XLT One-Step RT-qPCR ToughMix^®^, Low ROX™ (VWR Hannover, Lower Saxony, Germany) with the following cycling profile: 1 cycle 50 °C–10 min, 1 cycle 95 °C–1 min, and 45 cycles 95° C–10 sec, 60° C–40 sec. qPCR was performed by using the PerfeCTa qPCR Toughmix (2×) (without ROX) (Quanta) with the following cycle profile: 1 cycle 95 °C–10 min and 45 cycles 95 °C–15 sec, 60 °C–60 sec. QuantStudio3 Real-Time PCR Systems (Applied Biosystem, Thermo Fisher Scientific) was used to carry out amplification reactions. Samples were tested in duplicates per reaction. The data are presented as cycle threshold (CT) values, which were normalized against the 60S ribosomal protein L13 (RPL13) housekeeping gene of the same sample (*Δ*C*_T_*).

#### 2.3.3. Immunohistochemical (IHC) Staining

After fixation in 4% buffered formalin, TOCs (*n* = 5/group/time point) were embedded in paraffin wax. Sections (3 µm thick) were prepared and transferred to microscope slides (Superfrost Ultra Plus, Menzel B.V. & Co.KG, Braunschweig, Germany) and air-dried for 24 h. Paraffin was removed by Xylol and by descending alcohol concentrations. Following standard procedures, the antigen retrieval was performed by incubation in sodium citrate buffer overnight at 60 °C. Afterwards, cells were permeabilized with 0.2% Triton X (Merck, Darmstadt, Germany) in phosphate buffered saline (PBS) (Merck, Darmstadt, Germany). Subsequently, primary antibodies (Abs) were added and incubated overnight in a humid chamber at 4 °C. As primary Abs, we used rabbit anti *M. gallispeticum* [4] diluted 1:1000 and turkey anti AMPV A [21] diluted 1:300. The slides were then incubated with the secondary Abs for 45 min in a humid chamber at 36 °C. As secondary Abs, we used goat anti rabbit IgG (H+L) labeled with Alkaline Phosphatase (AP) (Biozol Eching, Germany), diluted 1:1000 and goat anti turkey (H+P) labeled with Horse Reddish Peroxidase (HRP) (Biozol), diluted 1:800. Staining was performed either with the VECTOR Red Alkaline Phosphatase Kit (Vector Laboratories Inc., Burlingame, CA, USA) or the DAB Peroxidase Substrate Kit (Vector Laboratories Inc.) to visualize the *M. gallispeticum* and AMPV antigens, respectively. The slides were counterstained with heamalaun and mounted with Aquatex (Merck, Darmstadt, Germany). Antigen detection was performed microscopically (Leica Microsystems, Germany) at a 400-fold magnification. Positive cells were counted in three microscopic fields per ring. The average number of positive cells of five rings per time point per treatment was calculated.

#### 2.3.4. Transmission Electron Microscopy

TOCs were fixed in 5% glutaraldehyde and further prepared at the Institute of Pathology, University of Veterinary Medicine, Hannover. The tracheal rings were cut into ultrathin sections and counterstained with uranyl acetate and lead citrate. Transmission electron microscopy was performed with a ZEISSEM10 transmission electron microscope (Zeiss, Germany) following standard procedures.

#### 2.3.5. Ciliostasis Assay

The ciliary activity was assessed daily via an inverted microscope. Tracheal rings were divided into 10 equal sections, equivalent to 10% of total ciliary activity per section. The average of the ciliary activity of 10 rings per time point per treatment was calculated.

#### 2.3.6. Histopathological Examination

TOCs were fixed in 4% formaldehyde and embedded in paraffin. 3µM-sections were obtained and transferred to microscope slides (corners cut, with frosted edge, Carl Roth^®^ GmbH + Co. KG) and air-dried for 24 h. The samples were stained with haematoxylin and eosin (H&E) according to standard methods.

#### 2.3.7. Determination of Cytokine mRNA Expression by RTqPCR

Primers and probes are listed in Table S1. RTqPCR was carried out using qScript™ XLT One-Step RT-qPCR ToughMix^®^, Low ROX™ (Quanta) and the following cycling profile: 1 cycle 55 °C-10 min, 1 cycle 95 °C-1 min and 40 cycles 95 °C-15 s, 57° C-45 s. The data are presented as the log 2-fold change in relation to the normalized data (*Δ*C*_T_*) of the non-inoculated control group. Data were normalized against the RPL13 housekeeping gene.

### 2.4. Statistical Analysis

Normal distribution was tested with the Shapiro–Wilk normality test. Statistically significant differences between investigated time points per group were evaluated with the Tukey HSD All-Pairwise comparisons test (ANOVA, with *α* = 0.05) and verified with the two-sample *t*-test by comparing two consecutive time points. Data not normally distributed between the inoculated and non-inoculated groups were evaluated by applying the Wilcoxon Rank Sum test. For multiple comparisons between the control, the mono- and the dual-inoculated groups per time point, the Kruskal-Wallis all-pairwise comparison test (*α* = 0.05) was used. All tests were conducted with Statistix, Version 10.0 (Analytical Software, Tallahassee, FL, USA). Differences were considered significant with a *p* value < 0.05.

## 3. Results

### 3.1. The Effect of the Inoculation-Scheme on M. gallispeticum and AMPV Replication

#### Quantification and Detection of Viral Colonization and Replication

**Figure 1:**
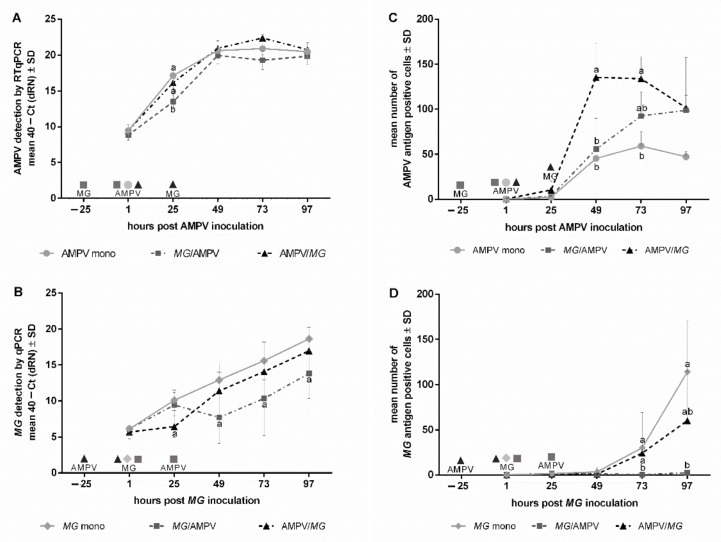
Effect of the inoculation scheme on AMPV and *M. gallispeticum* replication during the early phase post inoculation (pi) Five TOCs per group were taken at 1, 25, 49, 73, and 97 hpi (hpAMPVi and/or hp*MG*i) and processed for AMPV (**A**) and *M. gallispeticum (MG)* (**B**) quantification. Values were normalized against the RPL13 housekeeping gene. Normalized data are presented as mean 40–Ct. Samples of the same experimental setup were used for immunohistochemical staining of AMPV (**C**) and *M. gallispeticum* (**D**)**.** Antigen-positive cells were counted in three microscopic fields per TOC. The average of five rings per time point per group was calculated. Error bars indicate standard deviation (SD). Small letters indicate significant differences between different inoculation groups at the same time point. (**A**,**B**) Tukey HSD all-pairwise comparison test. (**C**,**D**) Kruskal–Wallis all-pairwise comparison test. *M. gallispeticum* indicates *M. gallispeticum* inoculation, AMPV indicates AMPV inoculation. Single symbols within the graph represent different inoculation time points. Graphs represent summarized data of two independent repeats of experiment.

To evaluate the early phase of pathogen colonization and replication, we focused on the period from 1 to 97 h post mono- or second pathogen inoculation. The viral load of the three AMPV-inoculated groups increased significantly from 1 to 49 hpAMPVi, followed by a plateau phase, as confirmed with both detection methods (*p* < 0.05) (Figure 1). Quantified by RTqPCR, a significant effect of the inoculation scheme on virus replication was detected at 25 hpAMPVi. The *M. gallispeticum*/AMPV group showed significantly lower AMPV-RNA quantities compared to the other two groups (*p* < 0.05). At 49hpAMPVi, the AMPV/*M. gallispeticum*-inoculated TOCs showed the highest AMPV load compared to the other two groups, which lasted up to 73 hpAMPVi.

### 3.2. Quantification and Detection of Bacterial Colonization and Replication

For most time points and both detection methods, the *M. gallispeticum* load increased significantly over the entire sampling period (Figure 1B/D) (*p* < 0.05). Compared to the mono group, which showed a continuous increase via qPCR, the AMPV/*M. gallispeticum* group showed a delayed *M. gallispeticum* replication up to 25 hp *M. gallisepticum*i (hp*MG*i), followed by a significant increase up to 97 hp *M. gallispeticum*i (Figure 1B) (*p* < 0.05). These results were partially confirmed by immunohistochemistry, showing a smaller increase in the number of *M. gallispeticum*-positive cells in the AMPV/*M. gallispeticum* group compared to the *M. gallispeticum* mono group. On the other hand, in the *M. gallispeticum*/AMPV group, the *M. gallispeticum* detection rate first increased significantly up to 25 hp*MG*i (*p* < 0.05), followed by a drop up to 49 hp*MG*i. This was followed by an increase up to 97 hp*MG*i. Interestingly, the *M. gallispeticum* antigen in the *M. gallispeticum*/AMPV group was not detectable until 73 hp*MG*i. Only few antigen-positive cells were detected in the *M. gallispeticum*/AMPV group 97 hp*MG*i, while the other groups expressed about 60–120 *M. gallispeticum*-positive cells/TOC on average (Figure 1D).

### 3.3. Ultrastructural Insights into Host–Pathogen Interactions after AMPV and M. gallispeticum Mono- and Dual-Infection

**Figure 2:**
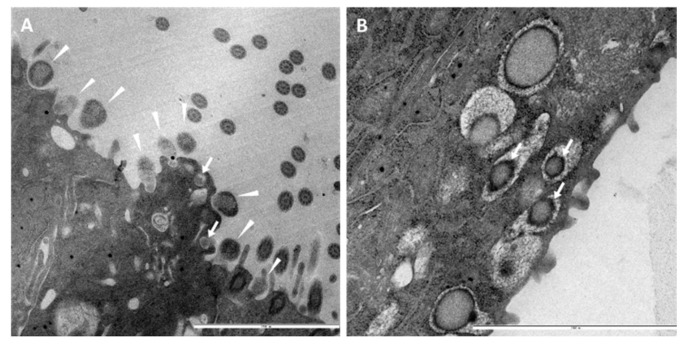
Ultrastructural insights into the adherence and colonization of *M. gallispeticum* at the epithelial surface of chicken TOC. Electron microscopic images of the epithelial surface of chicken TOCs inoculated with *M. gallispeticum* and/or AMPV. (**A**) *M. gallispeticum*-inoculated, 73 hp*MGi*, ×16,000 magnification, (**B**) *M. gallispeticum*/AMPV- inoculated, 25 hp*MGi*/1hpAMPVi, ×25,000 magnification. Five TOCs/group/time point were taken at 1, 25, 49, and 73 h post mono- or second inoculation. White arrowheads indicate attached and cell-invading mycoplasmas, and white arrows indicate intracellular located mycoplasma.

**Figure 3:**
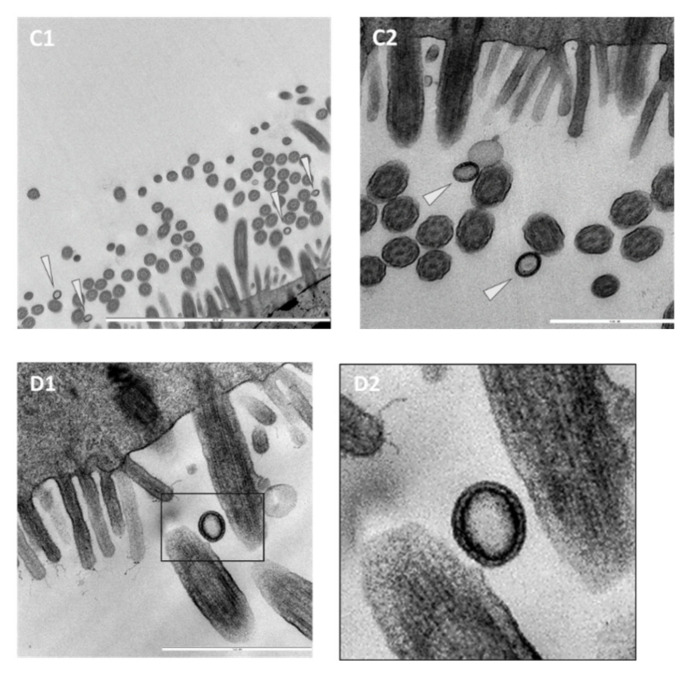
Ultrastructural insights into the adherence and colonization of AMPV at the epithelial surface of chicken TOC. Electron microscopic images of the epithelial surface of chicken TOCs inoculated with *M. gallispeticum* and/or AMPV. (**C1**) AMPV-inoculated, 15mpAMPVi, ×12,500 and (**C2**) ×40,000 magnification; (**D1**) AMPV-inoculated, 15mpAMPVi, ×50,000 magnification. Five TOCs/group/time point were taken at 1, 25, 49, and 73 h post mono- or second inoculation. Black-outlined arrowheads indicate virus-like particles. Black box indicates section on next image (**D2**).

Mono- and dual-inoculated TOCs were evaluated for extra- and intracellularly located bacteria and virus particles via positive contrast electron microscopy. Evaluation revealed extra- and intracellular mycoplasmas in the mono- and dual-inoculated groups (Figure 2A,B). At 73 hp*MG*i, *M. gallispeticum* adhering to and invading the apical cell border were detected in the *M. gallispeticum* mono group. Meanwhile, in the *M. gallispeticum*/AMPV group, mycoplasmas were already located intracellularly at 25 hp*MG*i (1hpAMPVi). No bacteria were detected in the control or the AMPV/*M. gallispeticum* group at any investigated time point. Virus-like structures attached to the cilia were detected in the AMPV mono group at 15 and 45 mpi (Figure 3C1–D2). Between 1 and 97 hpAMPVi, virus particles were no longer detected in any group.

### 3.4. The Effect of AMPV and M. gallispeticum Mono- and Dual-Infection on the Lesion Development

#### Ciliostasis Assay

**Figure 4:**
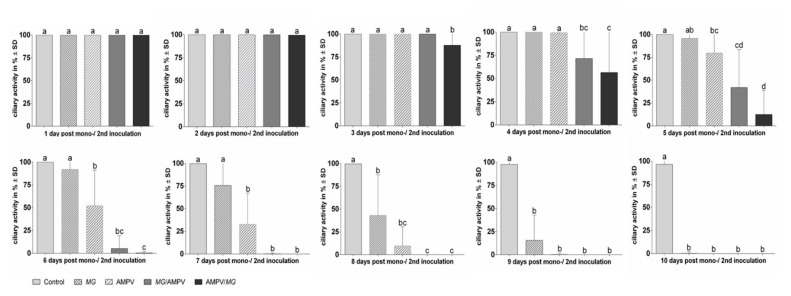
Progress of ciliostasis in chicken TOCs 1–10 days after inoculation with *M. gallispeticum* and/or AMPV. Graphs represent the average daily ciliary activity (in %) of 10 TOCs/group (Exp.1). TOCs were randomly allocated to five different inoculation groups: *M. gallispeticum* (*MG*) mono, AMPV mono, *M. gallispeticum*/AMPV (*MG*/AMPV), AMPV/*M. gallispeticum* (AMPV/*MG*)*,* and control. The TOCs were observed daily for ciliary activity up to 10 days post inoculation (dpi). Error bars indicate the standard deviation (SD). Letters indicate significant differences between the five different inoculation groups at the same time points, with *p* < 0.05, Kruskal–Wallis all-pairwise comparison test. Graphs represent the summarized data of two independent repeats.

On the first day post inoculation (dpi), all five groups showed 100% ciliary activity. At 4 dpAMPVi/3 dp*MG*i, the AMPV/*M. gallispeticum* group showed the first onset of ciliostasis compared to the other groups, which remained at 100% ciliary activity (Figure 4). In comparison to the mono-inoculated groups, the ciliary activity of the *M. gallispeticum*/AMPV group started to decrease significantly at 5 dp*MG*i/4 dpAMPVi (*p* < 0.05). Up to this time point, the mono-inoculated and control groups remained at 100% ciliary activity. The onset of ciliostasis in the mono-inoculated groups was observed at 5 dpi and 6 dpi, progressing faster in the AMPV group compared to the *M. gallispeticum* group. For both dual-inoculated groups, complete ciliostasis was observed 8 dpAMPVi/7 dp*MG*i (AMPV/*M. gallispeticum*) and 9 dp*MG*i/8 dpAMPVi (*M. gallispeticum*/AMPV). At 10 dpi, both mono-inoculated groups showed complete ciliostasis, while the control group remained at 100% ciliary activity throughout the observation period.

### 3.5. Histopathological Examination

**Figure 5:**
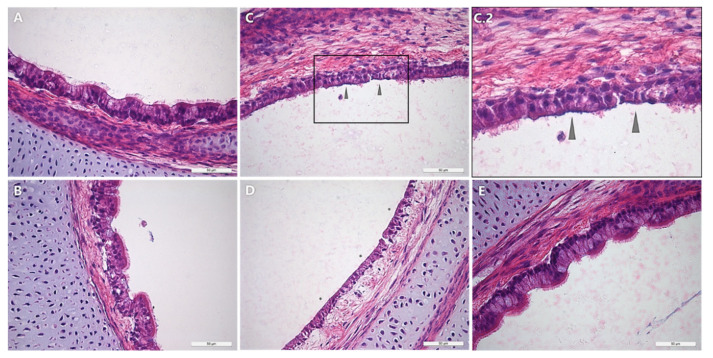
Histopathological examination of the lesion development at the epithelial surface of chicken TOCs with *M. gallispeticum* and/or AMPV. Microscopic images of the AMPV-, *M. gallispeticum*-, *M. gallispeticum*/AMPV-, AMPV/*M. gallispeticum*-inoculated and control groups. (**A**) 97 hpAMPVi, (**B**) 97 hp*MG*i, (**C.2**) 121 hp*MG*i/97 hpAMPVi, (**D**) 121 hpAMPVi/97 hp*MG*i, (**E**) non-inoculated control, all × 400 magnification. Gray arrows indicate partial ciliary destruction. Asterisks indicate total destruction of cilia. Black box indicates magnification of selected area with ciliary destruction. (**C**) 121 hp*MG*i/97 hpAMPVi.

To determine whether ciliostasis correlates with progressive ciliary destruction, inoculated TOCs were examined histopathologically for up to 97 h post mono- or second inoculation. The period of evaluation was limited due to the progressively negative impact on the epithelial integrity over time, leading to the loss of the epithelial layer during sample preparations. In the control group, the ciliary border remained intact until the last time point of evaluation (Figure 5). No significant ciliary loss was detected in the mono-inoculated groups. The cilia of the *M. gallispeticum*/AMPV group remained intact until 97 hp*MG*i/73 hpAMPVi but showed destruction at 121 hp*MG*i/97 hpAMPVi, correlating with early ciliostasis. Compared to the other dual-inoculated group, the ciliary loss in the AMPV/*M. gallispeticum* group started at 73 hpAMPVi/49 hp*MG*i and progressed until 121 hpAMPVi/97 hp*MG*i, resulting in complete ciliary loss.

### 3.6. The Effect of M. gallispeticum and AMPV Mono- and Co-Infection on Innate Immune Responses

#### Quantification of IFNα, iNOS, and IFNλ mRNA Expression

**Figure 6:**
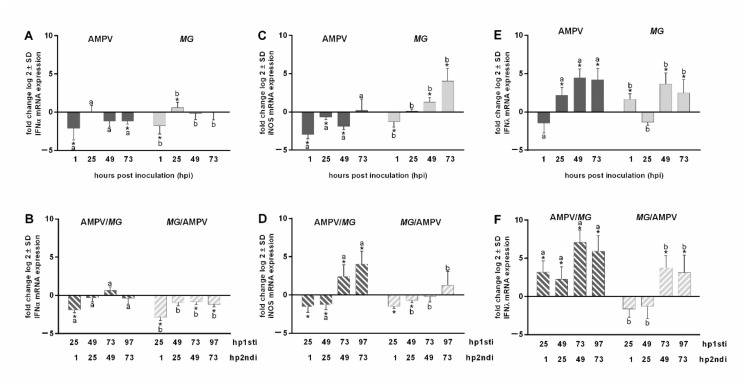
Quantification of IFNα (**A**,**B**), iNOS (C,D) and IFNλ (**E**,**F**) mRNA expression after AMPV and *M. gallispeticum* mono- and co-infection. TOCs were either mono- (**A**,**C**,**E**) or co-infected (**B,D,F**) with AMPV and *M. gallispeticum* (*MG*). Five TOCs/group/time point were taken. ΔCt values were normalized to the RPL13 RNA expressions and the log 2-fold change was calculated. The mRNA expressions are presented as log 2-fold change compared to the control groups. Error bars indicate the standard deviation (SD). Asterisks indicate significant differences between the mRNA expression levels of the inoculation group and the negative (pathogen-free) group at the same time point, with *p* < 0.05, Wilcoxon Rank sum test. Small letters indicate significant differences between two respective groups at the same time point (*p < 0.05*, Wilcoxon Rank sum test). Graphs represent the summarized data of two independent repeats.

The mRNA expression of IFNα, iNOS, and IFNλ was quantified (Figure 6). IFNα mRNA expression was mostly downregulated in both mono-inoculated groups throughout the sampling period (Figure 6A). Comparable results were observed for both dual-inoculated groups (Figure 6B), with the most significant downregulation in the *M. gallispeticum*/AMPV group 25 hp*MG*i/1 hpAMPV (*p* < 0.05). The iNOS mRNA expression was significantly downregulated in the AMPV mono group from 1 to 49 hpAMPVi, in contrast to the significant upregulation in the *M. gallispeticum* mono group 49 and 73 hp*MG*i (*p* < 0.05) (Figure 6C). In both dual-inoculated groups, iNOS showed a significantly downregulated mRNA expression 25 h post first inoculation (hp1^st^i) (equals 1 h post second inoculation (hp2^nd^i)) and 49 hp1^st^i (equals 25 hp2^nd^i), with *p* < 0.05 (Figure 6D). While iNOS was only slightly upregulated at 97 hp*MG*i/73 hpAMPVi in the *M. gallispeticum*/AMPV group, it showed a significant upregulation between 73 and 97 hpAMPVi/49 and 73 hp*MG*i (*p* < 0.05) in the AMPV/*M. gallispeticum* group. IFNλ was significantly upregulated both in the AMPV mono group between 25 and 73 hpAMPVi and in the *M. gallispeticum* mono group between 49 and 73 hp*MG*i (*p* < 0.05) (Figure 6E). The most significant upregulation of IFNλ, by 3–7-fold compared to the control groups, was observed in the AMPV/*M. gallispeticum* group at all investigated time points (*p* < 0.05) (Figure 6F). In the *M. gallispeticum*/AMPV group, the significant upregulation of the IFNλ mRNA expression was only detected at 73 and 97 hp*MG*i /49 and 73 hpAMPVi (*p* < 0.05). Interestingly, the early downregulation of IFNλ in both mono-inoculated groups at 1 hpAMPVi and 25 hp*MG*i was only confirmed in the *M. gallispeticum*/AMPV group (25 hp*MG*i/1 hpAMPVi). Meanwhile, the AMPV/*M. gallispeticum* group showed significant upregulation of IFNλ mRNA expression at all investigated time points (*p* < 0.05).

### 3.7. Correlation of Pathogen Replication and IFNα mRNA Expression 1 to 25 hpi

**Figure 7:**
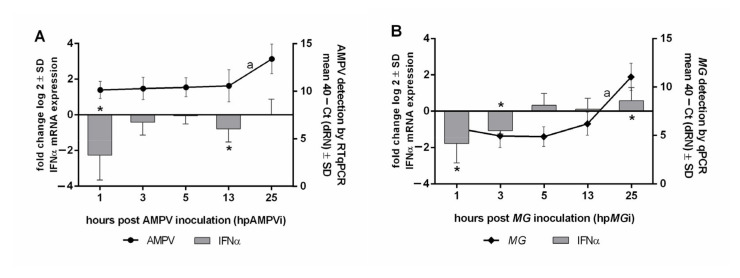
Quantification of AMPV and *M. gallispeticum* in correlation to the IFNα mRNA expression. (**A**) AMPV-inoculated group and (**B**) *M. gallispeticum* (*MG*)-inoculated group, with 10 samples/group/time point. TOC (*n* = 10/group/time point) were inoculated with *M. gallispeticum* and/or AMPV. Error bars indicate the standard deviations (SD). Asterisks indicate significant differences between the mRNA expression levels of the inoculated group and the control group at the same time point, with *p* < 0.05, two sample *t*-test. Letters indicate significant differences in the pathogen load between two consecutive time points (*p* < 0.05, Tukey HSD all-pairwise comparison test). Graphs represent the summarized data of two independently repeated experiments.

To investigate the very early innate immune response, we focused on the time period between 1 and 25 hpi to quantify the IFNα mRNA expression in correlation to the pathogen replication pattern (Figure 7). We were limited to mono-infections due to the time range of 24 h, which represents the usual time range between the first and second inoculation. A significant increase in AMPV and *M. gallispeticum* load was observed between 13 and 25 hpi (*p* < 0.05). IFNα expression was mostly downregulated at all investigated time points in the AMPV group, with very early significant downregulations compared to the control group at 1 and 25 hpAMPVi (*p* < 0.05) (Figure 7A). Compared to the AMPV group, IFNα was significantly downregulated in the *M. gallispeticum* group (*p* < 0.05) at 1 and 3 hp*MG*i (Figure 7B). A positive correlation between the increasing bacterial load and IFNα mRNA expression was shown over the complete observation, while a correlation between the viral load and IFNα expression level was only detected from 13 to 25 hpi.

## 4. Discussion

In our study, we wanted to examine how co-infections might affect the pathological impact on the host and whether the order of invading pathogens leads to changes in the host–pathogen–pathogen interaction. To determine the differences in pathogen replication after varying inoculation schemes, we quantified the pathogen load via (RT)qPCR and immunohistochemical staining. In the dual-inoculation groups, *M. gallispeticum* affected the replication pattern of AMPV depending on the order of infection. A subsequent inoculation with *M. gallispeticum* led to a prolonged increase in the viral load and number of AMPV antigen-positive cells, respectively. On the contrary, a significantly lower viral load and lower number of antigen-positive cells were observed in the *M. gallispeticum*/AMPV group. Our data provide circumstantial evidence that AMPV proliferation lasts longer due to subsequent infection with *M. gallispeticum*. Our study also clearly demonstrates that different pathogen detection methods that may provide variable results in detection rates due to differences in sensitivity as well as the detection of different stages of pathogen replication cycle (genome versus pathogen protein).

Preceding viral infections, predisposing for secondary bacterial infections was already described for other respiratory viruses such as influenza A virus, human rhinovirus, parainfluenza virus, and HMPV [51]. Viruses such as HMPV, the closest relative to AMPV [52], also benefit from bacterial pre-infections. Van de Zande et al. pre-infected human epithelial cells with *S. pneumoniae* which led to an increased susceptibility to HMPV infections [53]. They suggested mechanisms such as inhibition of ciliary beating, activation of immune cells, and stimulation of TLRs or immune evasive factors counteracting host immune responses and facilitating HMPV infection. Our study might be the first to demonstrate the beneficial effects of a subsequent bacterial infection to a preceding viral infection. The mechanisms contributing to our observations are comparable to the suggested mechanisms in the aforementioned study. Lipoproteins of mycoplasmas being recognized by TRLs stimulate the release of pro-inflammatory cytokines, their membrane lipids disrupt the lipid bilayer of the cellular membrane, and their immune evasion processes and the release of hydrogen peroxide led to host tissue damage [54,55]. In our study, the detected iNOS upregulation induced by *M. gallispeticum* may contribute to inflammatory processes and cell damage such as ciliary destruction [56,57]. This mechanism might facilitate the infection and replication of preceding AMPV.

Dual inoculation with AMPV also affected the infection process of *M. gallispeticum*. A pre-inoculation with AMPV led to a delayed *M. gallispeticum* replication. On the other hand, a subsequent inoculation with AMPV induced a significant reduction in the *M. gallispeticum*-genome detection rate and the number of *M. gallispeticum*-antigen positive cells compared to the *M. gallispeticum* mono-inoculated TOCs (*p* < 0.05). These findings suggest that the infection mechanisms of *M. gallispeticum* could be impaired by the presence of AMPV during the early phase of infection. We speculate that AMPV interferes with the ligand–receptor interaction of *M. gallispeticum*, which affects the bacterial attachment and gliding processes and subsequent cell invasion. Previous studies demonstrated that *Mycoplasma pneumoniae (M. pneumoniae)*, a close relative of *M. gallispeticum* [58], uses sialic acid-linked receptors to mediate initial attachment to epithelial cells. The researchers experimentally reduced the number of receptors, which led to a significant drop of mycoplasma attachment and movement to the apical cell border [59]. The movement (gliding) is accomplished by a postulated catch-pull-release cycle [60], which may be very vulnerable to interferences during the binding process [59]. A similar pattern can be assumed for *M. gallispeticum* since Glasgow et al., found that *M. gallispeticum* also interacts with sialyl glycoproteins to mediate attachment [61], and similar proteins required to initiate the infection process were found [58].

On the other hand, HMPV has two mechanisms to initiate infection. It uses the fusion protein (F) and the attachment protein (G) to bind to the cell membrane. While the F protein mediates direct binding to heparin sulfate proteoglycans to initiate the infection [62,63,64], there is some evidence that the G protein also uses sialic acid-containing glycosaminoglycan as co-receptors [65]. Therefore, we speculate that the interaction of AMPV and *M. gallispeticum* with sialic acid-linked receptors might overlap in the dual-inoculation groups in the early phase of infection and may thereby temporarily block the receptors for *M. gallispeticum* attachment and gliding. This effect on the initial infection has an impact on all subsequent processes in the host, which was demonstrated by our results of subsequent lesion development and innate immune response.

The observed differences in viral replication between the dual-inoculated groups affected the onset and progression of ciliostasis and subsequent ciliary destruction. With AMPV as the preceding pathogen, the ciliary activity started to decrease earlier compared to the *M. gallispeticum*/AMPV group. The observation of the ciliary destruction agrees with the different progressions towards ciliostasis in these groups. Comparable effects were demonstrated in field studies in turkeys. They found that the predisposing effect of AMPV on secondary infections with *E. coli* led to increased mortality rates [66,67], which confirms our results.

Another parameter significantly affected by the viral presence and the order of infection is the IFNλ mRNA expression. AMPV as the preceding pathogen led to a significant upregulation over the complete observation period, with the most significant induction of all examined immune response parameters observed in all groups. This clearly indicates a strong IFNλ response to an infection with AMPV. Previous studies described the IFNλ induction as a typical antiviral response, as confirmed by infections with respiratory or intestinal viruses in chickens [32,68]. Pott et al. also mentioned the possible inhibitory effect of IFNλ on viral replication of avian influenza virus and infectious bronchitis virus in chicken and human lung cells, respectively [69]. In our study, we clearly demonstrated a strong correlation between the presence of AMPV and IFNλ expression, as well as a potential inhibitory effect of IFNλ on the viral replication in the later phase of infection, indicated by the plateau phase in both detection tests at later time points.

Surprisingly, *M. gallispeticum* also affects the IFNλ mRNA expression. For IFNγ, *M. gallispeticum* has been described to induce a significant increase in the mRNA expression leading to macrophage activation and inflammatory processes in the host [57]. However, effects of *M. gallispeticum* on IFNλ expression levels have only been detected in turkeys. [4]. In our study, we demonstrated for the first time that *M. gallispeticum* inoculation also leads to upregulation of IFNλ mRNA expression in chickens. Thereby, *M. gallispeticum* replication is not affected by this immune response, as indicated by the continuous increase of the bacterial load after 25 and 49 hp*MG*i in the AMPV/*M. gallispeticum* and *M. gallispeticum*/AMPV group, respectively. Further studies are needed to confirm these expression studies also at the protein level, demonstrating biologically active immune mediators in the context of AMPV and *M. gallispeticum* mono- and co-infections.

To gain better insights into pathogen adherence and cell invasion pattern, we also performed electron microscopic investigations. Virus-like particles were detected between 15 and 45 mpAMPVi in the AMPV mono group, with no extracellular attached virus detected at later time points, confirming early cell invasion as suggested for HMPV [70]. Cox et al. experimentally demonstrated viral fusion with the cell membrane between 15 and 60 min, supporting our observations. Easton et al. described the fusion between the host cell membrane and the viral envelope, followed by release of the internal components into the cytoplasm [71]. Due to this process, the detection rate of intracellularly viral RNA by positive contrast electron microscopy is limited, which might explain why no intracellular-located virus was detected at later time points. To our best knowledge, AMPV has never been shown in the extracellular space. With our findings, we provide the first evidence of a very early cell invasion.

Images of *M. gallispeticum*-inoculated TOCs revealed intracellular mycoplasmas at 25 hp*MG*i. In vitro studies conducted with sheep and chicken erythrocytes demonstrated cell invasion of *M. gallispeticum* from 30 min to 8 hpi via gentamycin invasion assay, followed by a significant increase from 8 to 24 hpi [26]. They suggest that the delayed increase of intracellular bacteria might be caused by a proportional increasing replication rate in the early phase of infection and the subsequent invasion of further uninfected cells by next generations. We also detected adhering and cell invading *M. gallispeticum* 73 hp*MG*i, presumably representing a later generation, as confirmed by the increased bacterial load detected via qPCR and immunohistochemistry.

## 5. Conclusions

It is well known that infections with multiple pathogens can affect the course of the disease and lesion development in a host. In our study, we clearly demonstrate that dual infections affect the pathogen replication pattern and accelerate lesion development and host immune responses. The order of inoculating pathogens has a significant impact on both the primary and the secondary invading pathogen. Pre-infection with AMPV followed by *M. gallispeticum* as a secondary pathogen leads to a prolonged viral replication, a more significant innate immune response, and lesion development and causes a delay in the *M. gallispeticum* replication. Our study shows, for the first time, that *M. gallispeticum* replication may be impaired by a subsequent AMPV infection, possibly by blocking specific cell membrane receptors, leading to interferences in the bacterial attachment process. Subsequently, the innate immune response is less pronounced, and lesion development appears at a later point, compared to the reverse order of pathogen inoculation. Our study provides new insights into the host–pathogen–pathogen interaction of AMPV and *M. gallispeticum* in the chicken respiratory tract and may thereby provide better understanding of the infection and interaction mechanisms. However, further studies need to be conducted to gain more knowledge about the infection processes of both pathogens and how exactly they affect one another.

## Figures and Tables

**Table 1:** The Five Different Inoculation Schemes Used for Experiments and Investigated Parameters. All experiments were conducted with five inoculation groups. − = no pathogen, + = inoculation with the pathogen, +1 = inoculation with the first pathogen, and +2 = inoculation with the second pathogen, with a time difference between first and second inoculation of 24 h. Sampling time points: Exp. 1a and 2b => 1, 25, 49, 73, 97 h post second/mono inoculation (hp 2nd/m i); Exp. 1b and 3a => 1, 25, 49, 73 hp 2^nd^i; Exp. 1c => 1, 15, 30, 45 min post inoculation (mpi), 1, 3, 7, 13, 25, 49, 73 hp 2^nd^i and Exp. 3b => 1, 3, 5, 13 and 25 hp 2^nd^i. Exp. 2a daily microscopic observation for 10 dpi.

Groups	AMPV	*M. gallispeticum*	Experiment 1a, 1b, 1c	Experiment 2a, 2b	Experiment 3a, 3b
Control	−	−	Pathogen colonization and replication	Development of pathological lesions	Innate immune response
AMPV	+	−			
*M. gallispeticum*	−	+			
AMPV/*M. gallispeticum*	+1	+2			
*M. gallispeticum*/AMPV	+2	+1			

## Data Availability

Raw data will be provided upon request.

## Supplementary Materials

The following are available online at https://www.mdpi.com/article/10.3390/microorganisms9112407/s1, Table S1: Primer and probe sequences for pathogen and cytokine quantification.
