# Supplementary material for: New Insights into the Host–Pathogen Interaction of Mycoplasma gallisepticum and Avian Metapneumovirus in Tracheal Organ Cultures of Chicken
Source: Microorganisms. 2021 Nov 22;9(11):2407. doi: 10.3390/microorganisms9112407 (PMC8618481; doi:10.3390/microorganisms9112407)
Supplement: Supplementary file 1 [file microorganisms-09-02407-s001.zip › microorganisms-1431276-supplementary.pdf]

Table S1: Primer and probe sequences for pathogen and cytokine quantification

Target	Primer and probe[a]	Sequence[b] (5´- 3´)	Accession no.	Reference
**AMPV SH gene**	SH F<br>SH R<br>MB-SH P	TAGTTTTGATCTTCCTTGTTGC<br>GTAGTTGTGCTCAGCTCTGATA<br>(FAM)-CGCGATCATTGTGACAG CCAGCTTCACGATCGCG-(BHQ-1)	AB548428.1	Cecchinato *et al*., 2013
***MG* Lp gene**	Lp F<br>Lp R<br>Lp P	ACTAGAGGGTTGGACAGTTATG<br>RYAACATATCAGAAGCAGTCATT<br>(FAM)-CCCAACAACTTATCATCA GATTCTGGTGGT-(BHQ-1)	AY556071[d]	Ferguson *et al*., 2005
**RPL13**	RPL13 F<br>RPL13 R<br>RPL13 P	GGAGGAGAAGAACTTCAAGGC<br>CCAAAGAGACGAGCGTTTG<br>(FAM)-CTTTGCCAGCCTGCGCAT C-(BHQ-1)	NM_204999.1	Sewada *et al*.- 1996
**chIFNα**	chIFNα F<br>chIFNα R<br>chIFNα P	GACAGCCAACGCCAAAGC<br>GTCGCTGCTGTCCAAGCATT<br>(FAM)-ACTGATCTTTGCTGCCAA ACAGGC-(BHQ-1)	U07868.1[c]	Petersen *et al*., 2013
**chiNOS**	chiNOS F<br>chiNOS R<br>chiNOS P	CAACAGGAACCTACCATCTGAC<br>GACCACTGGATTCTCCCAATAC<br>(FAM)-ACTGATCTTTGCTGCCAAA CAGGC-(HBQ-1)	NM_204961	Hartmann *et al*., 2015
**chIFNλ**	chIFNλ F<br>chIFNλ R<br>chIFNλ P	CATCAGCCCTCTGGGAAAC<br>CTTGGAAGATGTGGAGGATGG<br>(FAM)-ACAGCCAAGAAGAAGGAG ACCGC-(BHQ-1)	KJ206897.1[c]	Sid *et al*., 2017

[a] F, forward primer; R, reverse primer; P, probe
[b] FAM, 6-carboxyfluorescein; BHQ-1, Black Hole Quencher® (without native fluorescence)
[c] Genomic DNA sequence from GenBank
[d] Target gene used for primer design
